# A common variant of the pregnancy-associated plasma protein-A (PAPPA) gene encodes a protein with reduced proteolytic activity towards IGF-binding proteins

**DOI:** 10.1038/s41598-019-49626-8

**Published:** 2019-09-13

**Authors:** Jane Alrø Bøtkjær, Pernille Rimmer Noer, Claus Oxvig, Claus Yding Andersen

**Affiliations:** 1 Laboratory of Reproductive Biology, The Juliane Marie Centre for Women, Children and Reproduction, Rigshospitalet, Copenhagen University Hospital, Copenhagen University, Copenhagen, DK-2100 Denmark; 2 Department of Molecular Biology and Genetics, University of Aarhus, Aarhus, DK-8000 Denmark

**Keywords:** Proteases, Glycoproteins, Mutagenesis, Molecular medicine

## Abstract

Pregnancy-associated plasma protein-A (PAPP-A) is a key regulator of insulin-like growth factor (IGF) bioactivity, by releasing the IGFs from their corresponding IGF-binding proteins (IGFBPs). The minor allele of the single nucleotide polymorphism (SNP), rs7020782 (serine < tyrosine), in *PAPPA* has previously been associated with recurrent pregnancy loss as well as with significant reduced levels of PAPP-A protein in human ovarian follicles. The aim of the present study was to reveal a possible functional effect of the rs7020782 SNP in *PAPPA* by comparing recombinant PAPP-A proteins from transfected human embryonic kidney 293 T cells. The proteolytic cleavage of IGFBP-4 was shown to be affected by the rs7020782 SNP in *PAPPA*, showing a significantly reduced cleavage rate for the serine variant compared to the tyrosine variant (p-value < 0.001). The serine variant also showed a trend towards reduced cleavage rates, that was not significant, towards IGFBP-2 and IGFBP-5 compared to the tyrosine variant. No differences were found when analysing cell surface binding, complex formation between PAPP-A and STC2 or proMBP, nor when analysing STC1 inhibition of PAPP-A-mediated IGFBP-4 cleavage. Regulation of IGF bioactivity in reproductive tissues is important and the rs7020782 SNP in *PAPPA* may disturb this regulation by altering the specific activity of PAPP-A.

## Introduction

The insulin-like growth factor (IGF) signalling pathway is involved in several biological systems including the human reproductive system^1–6^. The two ligands, IGF-1 and -2, show high structural similarity to proinsulin and they interact and transduce their signal through the IGF-1 receptor and the insulin receptor with different affinities^7,8^. Strict regulation of IGF bioactivity is crucial since abnormal levels of free IGFs in serum is sufficient to induce severe hypoglycaemic effects^9^. IGF bioactivity is antagonized by six different IGF binding proteins (IGFBPs), which bind the IGFs with equal or higher affinity than their receptors and thus prevent cellular signalling. The IGFBPs also function to prolong the short half-life of IGFs in the circulation and direct them to their target receptor^9–11^. Different IGFBP proteinases exist, which release the IGFs from the IGFBPs by proteolysis^12–14^. The metalloproteinase pregnancy-associated plasma protein-A (PAPP-A) is able to cleave IGFBP-2, IGFBP-4, and IGFBP-5^15,16^. Proteolytic cleavage of IGFBP-4 by PAPP-A strictly depends on the binding of IGFs, whereas, PAPP-A cleavage of IGFBP-5 is slightly inhibited when bound to IGFs^16^. Originally, PAPP-A was detected in serum from pregnant women, where it circulates in complex with the proform of eosinophil major basic protein (proMBP), which functions as an inhibitor of the proteolytic activity of PAPP-A^17,18^. Currently, PAPP-A is used as a biomarker of foetal aneuploidies in pregnancies^17^. Two other inhibitors of PAPP-A, stanniocalcin-1 and -2 (STC1 and STC2), were recently discovered^19–21^. STC1 has been identified as a proteinase inhibitor towards PAPP-A forming noncovalent high-affinity complexes^19,20^. In contrast, STC2 binds covalently to PAPP-A^21^. Thus, the STCs indirectly inhibit IGF signalling by counteracting PAPP-A activity, adding another level of regulation to the IGF system.

Previous studies have identified different essential protein modules in PAPP-A^22–27^. The C-terminal part of PAPP-A contains five complement control protein (CCP) modules. The CCP modules mediate adhesion of PAPP-A to the cell surface through glycosaminoglycans, thereby locating the release of the bioactive IGFs close to their corresponding receptors^26,27^.

A recent study found two inactivating mutations in PAPP-A2, a homolog of PAPP-A, in two independent families with growth failure and markedly lower free IGF-I levels^5^. In addition, carriers of a rare missense variant in the STC2 gene (rs148833559) are approximately 2.1 cm taller compared to non-carriers^28^. Functional *in vitro* studies revealed that the ability of this variant to bind and inhibit PAPP-A-mediated IGFBP-4 cleavage was reduced, suggesting higher levels of bioactive IGFs that promote growth^28^. These earlier studies highlight the tremendous phenotypic effect small genetic variations may have. Previous studies have investigated single nucleotide polymorphisms (SNPs) in the *PAPPA* gene and one specific SNP in *PAPPA*; rs7020782, has been associated with increased risk of recurrent pregnancy loss, risk of gestational diabetes mellitus, risk of developing carotid plaques, and risk for ischemic cerebrovascular disease^29–32^. Additionally, a recent study revealed a significant effect of this SNP on the level of PAPP-A protein in human ovarian follicles and suggested a possible adverse effect on IGF bioactivity^33^. The rs7020782 SNP is located in exon 14 of the *PAPPA* gene in the CCP-1 module and causes a change in the amino acid sequence (i.e. tyrosine >serine). Furthermore, the glycosylation of these two variants may differ because the site of variation encodes a potential N-glycosylation motif^22,23^ and may hereby affect the properties of the protein, such as ability to bind substrates.

The frequency of the homozygous genotype of the minor allele (the CC genotype: serine variant) of the rs7020782 SNP is reported to be approximately 7–12% in Western and Asian populations^34^.

The present study aims to define a possible functional effect of the rs7020782 SNP in *PAPPA* by comparison of the recombinant protein variants. Throughout this paper we will address the two rs7020782 SNP variants as: the tyrosine variant and the serine variant.

## Results

### Surface binding of PAPP-A is not affected by the rs7020782 SNP

**Figure 1:**
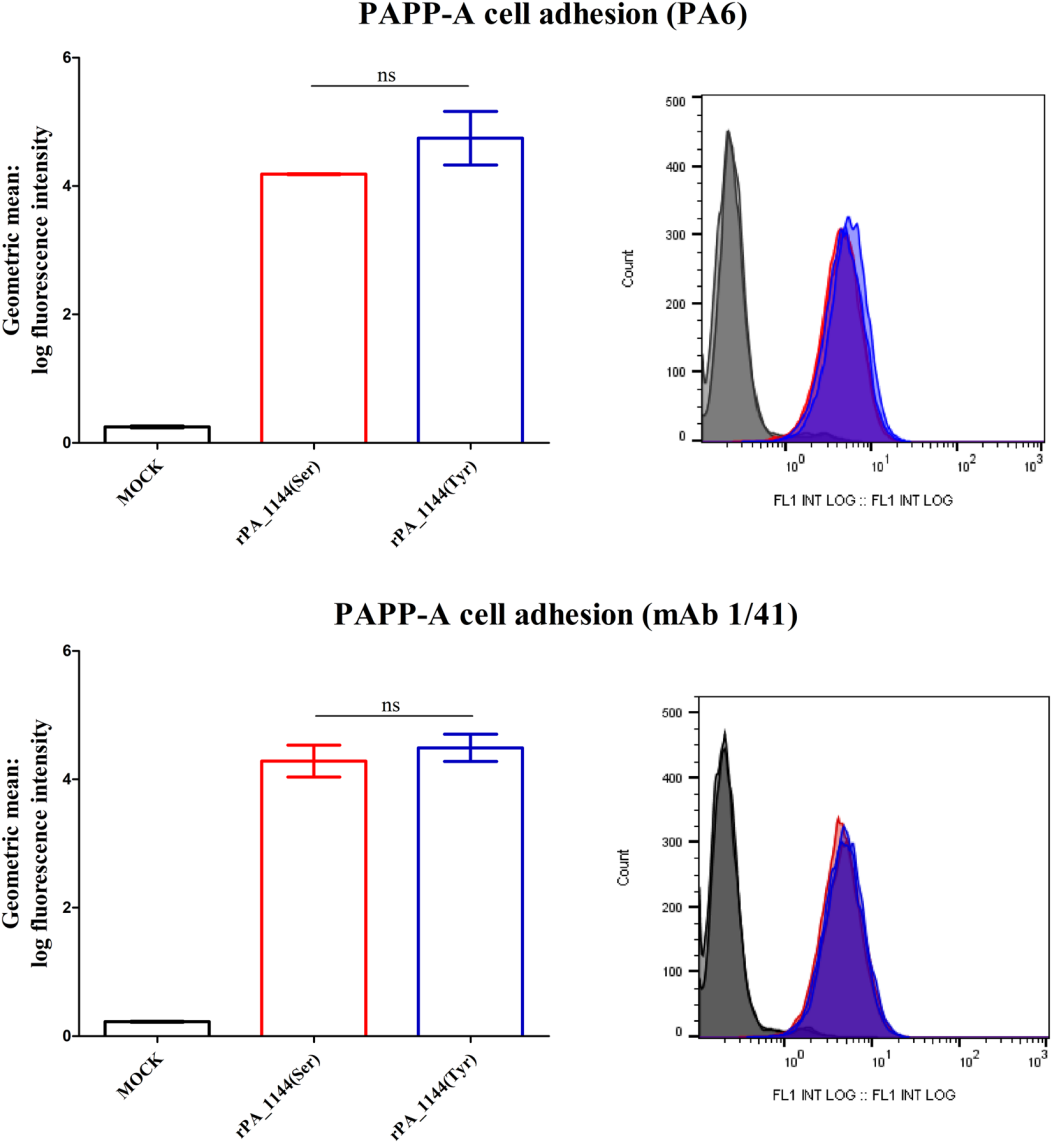
Detection of surface-bound PAPP-A by flow cytometry. Data are geometric mean (±SEM) of the log fluorescence intensity measured on a flow cytometer. Data are presented as mean values of the fluorescence intensity measured from HEK293T cells incubated with supernatants containing either the serine variant (rPA_1144(Ser): red bars) or the tyrosine variant (rPA_1144(Tyr): blue bars) of the rs7020782 PAPP-A SNP, or with supernatants with no PAPP-A (MOCK: black bars). Experiments were completed in triplicate and tested with two different primary antibodies against PAPP-A (Upper panel: PA6 antibody and lower panel: mAb 1/41 antibody). No significant difference (ns) was observed between the two PAPP-A variants.

Cell surface binding of PAPP-A to human embryonic kidney 293 T (HEK293T) cells was assessed by flow cytometry and the geometric mean of fluorescence intensities was analysed (Fig. 1). No statistically significant (ns) difference was observed between HEK293T cells incubated with supernatant containing the serine variant (rPA_1144(Ser)) and HEK293T cells incubated with supernatant containing the tyrosine variant (rPA_1144(Tyr)) (Fig. 1). HEK293T cells incubated with supernatant without PAPP-A (MOCK) showed background fluorescence. Experiments were completed in triplicate and tested with two different primary antibodies against PAPP-A.

### Complex formation between PAPP-A and its inhibitors: STC2 and proMBP

**Figure 2:**
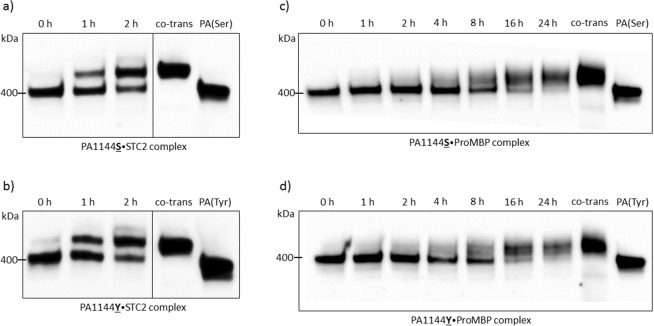
Complex formation of PAPP-A and STC2 or proMBP over time. Supernatants containing PAPP-A with either the serine (PA1144**S**) or the tyrosine (PA1144**Y**) variant of the rs7020782 SNP were incubated with STC2 (2a and 2b) or proMBP (2c and 2d) and formed covalent complexes over time (PAPP-A was incubated with STC2 or proMBP for 0, 1, 2, 4, 8, 16, and 24 hours). These protein complexes were visualized with Western blotting using a primary antibody against PAPP-A. A high molecular-weight band appears when complexes are formed (>400 kDa PAPP-A dimer). This figure displays PAPP-A incubated with STC2 from 0 to 2 h solely, since most PAPP-A has complexed with STC2 by this time-point and a potential difference would therefore be visible. Full-length blots and replications are presented in Supplementary Figs 2.1, 2.2. Supernatants from HEK293T cells co-transfected with both PAPP-A and STC2 or PAPP-A and proMBP were included in the Western blot as positive controls (co-trans) showing complexes exclusively. Supernatants from HEK293T cells transfected with PAPP-A alone (Serine variant: PA(Ser) or tyrosine variant: PA(Tyr)) was included as negative controls showing PAPP-A dimers solely. No difference was observed between the two PAPP-A variants in this Western blot analysis.

Complex formation between PAPP-A and STC2 or proMBP was studied by Western blot analysis. Supernatants from HEK293T cells transfected with the two rs7020782 PAPP-A variants incubated with supernatant containing STC2 (Fig. 2a,b) or proMBP (Fig. 2c,d) were examined. This analysis was performed to examine if the rs7020782 SNP in *PAPPA* influenced the covalent binding of PAPP-A to STC2 or proMBP.

Both STC2 and proMBP showed complex formation with the two different rs7020782 variants over time. These complexes were identified by a high molecular weight bands on the Western blot above the bands showing PAPP-A dimers (400 kDa). No difference was revealed with this Western blot analysis (Fig. 2).

Supernatants from HEK293T cells co-transfected with PAPP-A and STC2 (Fig. 2a,b: ‘co-trans’) or PAPP-A and proMBP (Fig. 2c,d: ‘co-trans’) were used as positive controls showing high molecular weight bands representing complete complex formation between PAPP-A and STC2 or proMBP. Supernatants from HEK293T cells transfected with PAPP-A alone (Serine variant: PA(Ser) or Tyrosine variant: PA(Tyr)) showed bands representing PAPP-A dimers solely at 400 kDa.

When incubating the two rs7020782 PAPP-A variants with supernatant from transfected HEK293T cells with empty vector (MOCK) no complex formation was observed (Supp. Fig. 2.1e). Experiments were completed in duplicate for both STC2 and proMBP.

### Cleavage rate of IGFBP-2, -4, and -5 was lower for the serine variant compared to the tyrosine variant of the rs7020782 SNP in *PAPPA*

**Figure 3:**
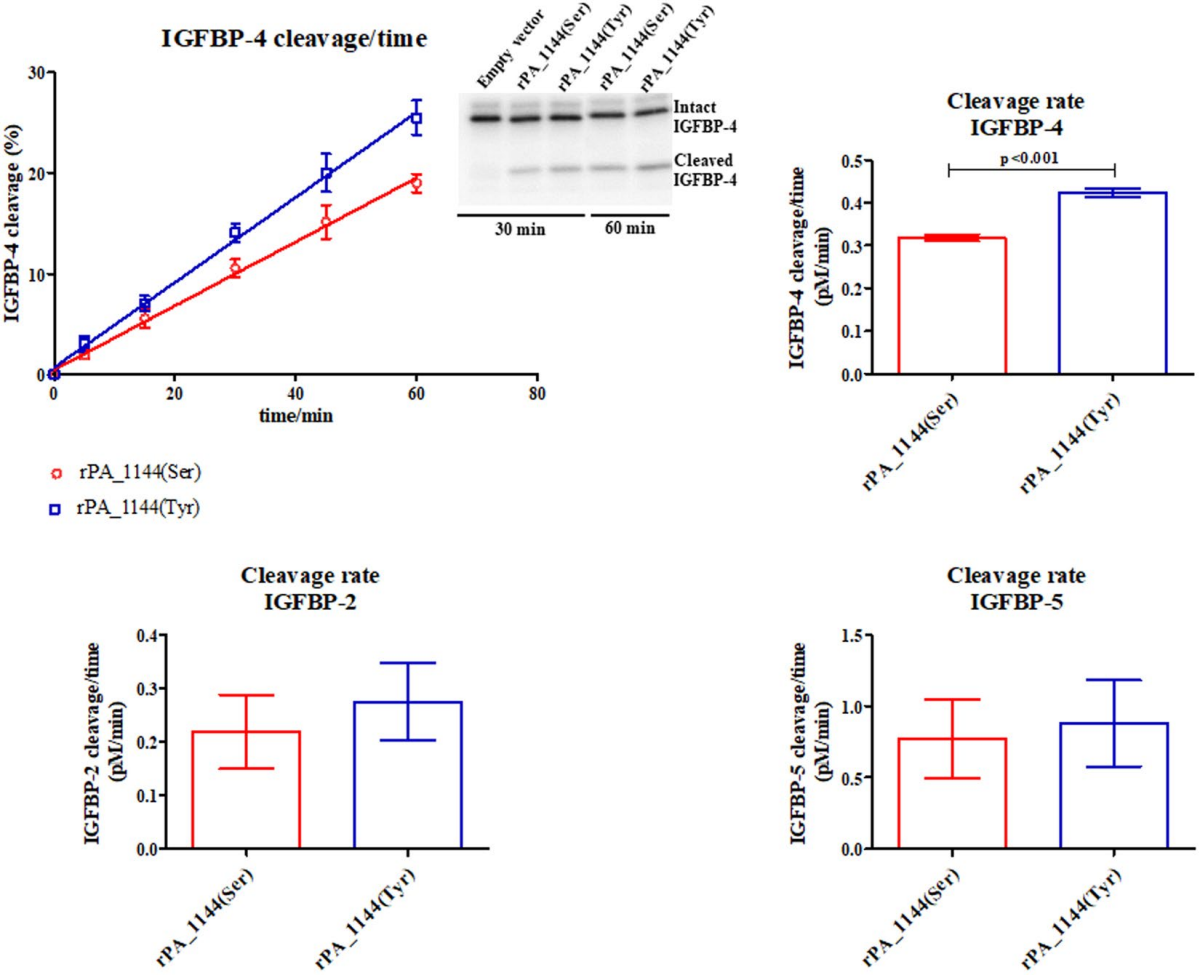
Proteolytic activity of the two rs7020782 PAPP-A variants towards radiolabelled IGFBP-2, IGFBP-4, and IGFBP-5. Supernatants from HEK293T cells transfected with the two PAPP-A rs7020782 variants (serine: red, tyrosine: blue) were incubated with IGFBP-2 (lower panel), IGFBP-4 (upper panel), or IGFBP-5 (lower panel). Cleavage reactions were stopped at different time-points and subsequently, intact and cleaved IGFBPs were quantified (insert). IGFBP-4 cleavage in percent versus time (linear regression) as well as cleavage rates (mean ± SEM) for all three binding proteins are shown. Results showing IGFBP-4 proteolysis are derived from four independent proteinase assays and with supernatants from four independent transfections. Results showing IGFBP-2 and IGFBP-5 proteolysis are derived from three independents assays. A significantly lower IGFBP-4 cleavage rate was observed for the serine variant of the rs7020782 SNP compared to the tyrosine variant (p-value < 0.001). Reduced IGFBP-2 (p-value = 0.60) and IGFBP-5 (p-value = 0.78) cleavage rates that was not significant were observed for the serine variant compared to the tyrosine variant.

Proteolytic activity towards IGFBP-2, IGFBP-4, and IGFBP-5 was assessed in order to examine whether the activity of PAPP-A was different between the two rs7020782 variants (Fig. 3).

Both rs7020782 variants of PAPP-A cleaved the three radiolabelled IGFBPs resulting in co-migrating cleavage products. Four independent proteinase assays revealed that the serine variant (rPA_1144(Ser)) presented a significantly lower cleavage rate of radiolabelled IGFBP-4 compared to the tyrosine variant (rPA_1144(Tyr)) (p-value < 0.001) (Fig. 3, upper panel). Furthermore, the results showed a trend towards reduced cleavage rates of radiolabelled IGFBP-2 and IGFBP-5 (Fig. 3, lower panel) for the serine variant compared to the tyrosine variant, which were derived from three independent proteinase assays.

### STC1 binds and inhibits the proteolytic activity of both PAPP-A rs7020782 variants

**Figure 4:**
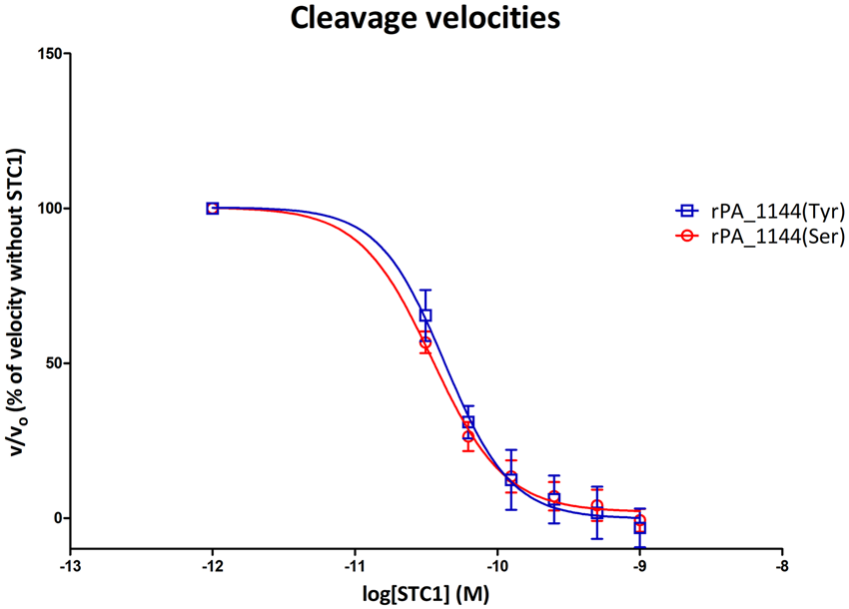
STC1 inhibition of PAPP-A-mediated IGFBP-4 cleavage. Relative initial velocities (v/v_0_) of PAPP-A-mediated IGFBP-4 cleavage (mean ± SEM) are plotted against logarithmic values of STC1 (M). Velocities are calculated from 3 time-points (15 min, 30 min, and 45 min) for each rs7020782 variant in the presence of increasing concentrations of STC1 (0.001 nM, 0.0312 nM, 0.0625 nM, 0.125 nM, 0.25 nM, 0.5 nM, and 1.0 nM) relative to the velocity without STC1. Data are fitted to a non-linear regression curve using ‘log(inhibitor) vs. response – variable slope’ in GraphPad Prism 5.0. The serine variant of the rs7020782 SNP is displayed in red and the tyrosine variant in blue. No significant difference was observed.

Cleavage of radiolabelled IGFBP-4 by the two PAPP-A rs7020782 variants with increasing concentrations of STC1 was studied in order to evaluate a possible effect on STC1 inhibition (Fig. 4). The proteolytic activities of both PAPP-A rs7020782 variants were inhibited by STC1 showing decreasing cleavage rates with increasing concentrations of STC1. No significant difference was detected in this assay.

## Discussion

PAPP-A-induced IGF signalling has shown to be dependent and regulated by various factors, and these should be addressed when studying the functionality of PAPP-A. First, cell surface adherence of PAPP-A through the CCP modules facilitates IGF receptor activation by releasing bioactive IGFs close to their receptors on the target cell. Second, proMBP and STCs ensure strict regulation of PAPP-A by inactivating PAPP-A-mediated proteolysis of IGFBPs and thereby downregulate IGF signalling. Thus, when evaluating PAPP-A functionality it is important to address the ability of PAPP-A to bind cell surfaces and the ability of PAPP-A to form complexes with the inhibitors in combination with studying its proteolytic activity.

This study provides a detailed analysis of potential functional effects of the rs7020782 SNP in the *PAPPA* gene using recombinant proteins from transfected HEK293T cells. The proteolytic activity of PAPP-A towards IGFBP-4 was significantly reduced for the serine variant of the rs7020782 SNP compared to the tyrosine variant (Fig. 3). Since PAPP-A acts as a crucial component in regulating IGF bioactivity, we suggest that the rs7020782 SNP disturbs this regulation.

IGFBP-4 exhibits complex interaction with PAPP-A by interacting directly with substrate-binding exosite(s) in the LNR3 module of PAPP-A^35^. The rs7020782 SNP in *PAPPA* may disturb this proteinase/substrate interaction and consequently result in the observed reduced proteolytic cleavage. In contrast, proteolysis of IGFBP-5 and IGFBP-2 by PAPP-A are not known to rely on specific interactions like IGFBP-4 and this may be the reason why the proteolytic cleavage of IGFBP-2 and IGFBP-5 only showed a trend towards reduced cleavage rates (not significant) for the serine variant of the rs7020782.

This study did not find any effect of the rs7020782 SNP on PAPP-A cell surface binding nor on the complex formation with the inhibitors proMBP and STC2. Additionally, the ability of STC1 to inhibit PAPP-A-mediated IGFBP-4 cleavage was not shown to be affected by the rs7020782 SNP.

As aforementioned, the rs7020782 SNP is located in exon 14 of *PAPPA* in the first CCP module^22,23^. Furthermore, only the serine variant allows for a potential N-glycosylation motif in the protein sequence at residue Asn-1142, however, when the tyrosine variant is present the potential N-glycosylation motif is lost^22,23^. This could potentially exert a significant effect on the protein conformational structure and thereby modulate the proteolytic specificity of PAPP-A. We did not observe an effect of the rs7020782 SNP on the adhesion of PAPP-A to HEK293T cells, thus, the CCP modules do not seem to depend on this site for cell adhesion.

The serine variant of the rs7020782 SNP in *PAPPA* has previously been associated with various negative physiological outcomes such as recurrent pregnancy loss^29^. Women carrying the rs7020782 SNP in *PAPPA* encoding the serine variant showed a tendency to have an increased risk of recurrent pregnancy loss, and a significantly increased risk of having at least one pregnancy loss after nine weeks of gestation^29^. Since the current study showed that the serine variant was associated with reduced IGFBP proteolysis, women carrying this variant might present with an altered IGF regulation and thereby a limited amount of free IGF to initiate signalling. Thus, the observed negative outcome on pregnancy is possibly affected by an altered IGF bioactivity through PAPP-A, most likely in combination with other genetic variations and aberrant levels of essential hormones.

In addition, adverse pregnancy outcomes have been associated with low levels of PAPP-A, for example an increased risk of intrauterine growth restriction, extremely premature delivery, preeclampsia, and stillbirth^36^. These studies highlight that the PAPP-A/IGF axis has an important function for pregnancy outcome.

A recent study displays a significant effect of the rs7020782 SNP *in vivo*^33^. Women carrying the homozygous CC genotype of the rs7020782 SNP (encoding the serine variant) displayed significantly lower concentrations of PAPP-A in follicle fluid (FF) of ovarian antral follicles compared to women carrying the major A allele (encoding the tyrosine variant). Additionally, reduced cleavage of radiolabelled IGFBP-4 was observed when incubated with FF from women with the serine variant, which was suggested to be explained by low PAPP-A levels. A tendency towards reduced estradiol levels and increased androgen levels in FF from women with the serine variant was also found, which was suggested to be influenced by an aberrant IGF regulation.

Studies of cultured human cumulus cells isolated from women undergoing fertility treatment show importance of FSH together with IGF-1 receptor activity for maximal stimulation of estradiol production, further underlining the significance of IGF signalling for ovarian steroid production^37^.

Overall, the newly found effects of the rs7020782 SNP on PAPP-A proteolytic activity may impact on both human ovarian function and pregnancy outcome.

The observed significant effect of the rs7020782 SNP on PAPP-A-mediated IGFBP-4 cleavage showed an approximately 1.5-fold reduction. Whether this reduction will have biological influence cannot be concluded from the current study, but a recent study showed reduced cleavage of radiolabelled IGFBP-4 when incubated with ovarian FF from women carrying this SNP^33^, which supports this notion. However, the specific activity of PAPP-A was not assessed.

Previous studies have investigated the effect of assisted reproductive technology (ART) on first trimester screening parameters such as PAPP-A^38,39^. Significant lower PAPP-A serum levels have been found in women receiving IVF/ICSI treatment compared to women conceiving naturally. Furthermore, the low levels of PAPP-A were significantly correlated with high estradiol levels at ovulation induction^39^. This suggests a regulative role of the hormones produced in the ovary prior to conception on the PAPP-A/IGF axis. In addition, IVF and ICSI are known to result in a higher risk of low birth weight compared to normal pregnancies^38,40^. This is suggested to be affected by PAPP-A/IGF in a study showing that the combination of slow early foetal growth and low PAPP-A lead to an approximately six-fold increased risk of having a small for gestational age infant^40^. Thus, the lower level of PAPP-A in ART associated with high estradiol levels could potentially impact the growth and further development of the foetus due to an abnormal IGF signalling.

Furthermore, women receiving IVF treatment and classified as poor responders for recombinant FSH have been shown to have significantly lower ovarian FF levels of IGF-1 together with lower serum and ovarian FF levels of oestradiol and progesterone^41^. Furthermore, this study found a positive correlation between total oocytes retrieved and ovarian FF levels of IGF-1 and suggests that the observed decreased level of IGF-1 in poor responders impairs the ovarian steroid production.

The minor allele of the investigated rs7020782 SNP (serine variant) appears to have potentially adverse effects on reproduction but persists at non-negligible frequencies. A potential explanation is that carrying a single copy of the minor allele may either have no phenotypic effect or, in some cases, even lead to potential beneficial effects, in for instance, breast and ovarian cancer where PAPP-A has shown to have tumour-promoting properties^42^. Alternatively, the minor allele may only show adverse effects in a certain genetic background, as, for instance, in Sub-Saharan Africa where the serine allele of the rs7020782 SNP instead of being the minor allele has been reported to be the major allele, which is exactly opposite to European and Asian populations^34^.

Besides recurrent pregnancy loss the rs7020782 SNP in *PAPPA* also associates with the risk of gestational diabetes mellitus, risk of developing carotid plaques, and risk for ischemic cerebrovascular disease^30–32^. An altered IGF bioactivity may be present in these cases, because of the reduced proteolysis of IGFBPs by PAPP-A, however, none of these studies included examinations of PAPP-A activity.

This study shows, for the first time, a direct link between the rs7020782 SNP in *PAPPA* and reduced proteolytic function, specifically towards IGFBP-4, which may be explained by reduced proteinase/substrate interaction. This may partly explain some of the mechanisms behind the different diseases associated with the SNP and highlights the importance of the IGF pathways in different biological aspects.

We suggest that the rs7020782 SNP in *PAPPA* could be used as a biomarker for identifying women with high risk of adverse pregnancy outcomes such as recurrent pregnancy loss and perhaps this could even allow for risk-specific treatment and improve the outcomes for these patients, however, further studies are needed to apply this in clinical settings.

## Materials and Methods

### Mutagenesis

Plasmids encoding human PAPP-A with the tyrosine variant of the rs7020782 SNP were constructed with the QuickChange II site-directed mutagenesis kit (Stratagene) using the primers:

5′-ctggctgtggagaatgcttatctcaattgctccag-3′ (forward primer)

5′-ctggagcaattgagataagcattctccacagccag-3′ (reverse primer)

(the rs7020782 change is underlined in the primer sequences).

Human PAPP-A plasmid (pcDNA3.1-PAPP-A) was used as a template described previously^18,27^. The PAPP-A constructs were verified with sequencing. The rs7020782 PAPP-A variant that encodes the serine variant was present in the pcDNA3.1-PAPP-A plasmid.

### Cell culture and transfection

HEK293T cells were maintained in high glucose Dulbecco’s modified Eagle’s medium supplemented with 10% foetal bovine serum, 2 mM glutamine, nonessential amino acids, and gentamicin (Invitrogen). Cells were plated in 6-cm tissue culture dishes and transfected with plasmid constructs containing cDNA encoding: human PAPP-A with the serine variant of the rs7020782 SNP, human PAPP-A with the tyrosine variant of the rs7020782 SNP, human proMBP (Human placental oligo-dT-primed cDNA encoding proMBP was used as a template^43^) and human STC2 (STC2 cDNA in the pcDNA3.1/Myc-His(−) was used as a template^21^). HEK293T cells transfected with empty vector were included as controls (MOCK). Transfection of cells was performed by calcium phosphate co-precipitation using 10 µg of plasmid DNA as previous described^21^. Media were harvested 24 hours and 48 hours post-transfection, cleared by centrifugation, and stored at -20 °C until further analysis.

### Enzyme-linked immunosorbent assay (ELISA)

The concentration of recombinant PAPP-A in the supernatants from the transfected HEK293T cells was determined by ELISA (picoPAPP-A, AL-101-i, Ansh Labs, Texas, USA) according to the manufacturer’s instructions (PBS + 1% BSA was used for dilution). The ELISA measures were used to achieve equal concentration of the two PAPP-A rs7020782 variants in the different functional assays.

### Flow cytometry

HEK293T cells were used for analysing cell surface adhesion of the PAPP-A rs7020782 variants^27^. Non-transfected cultured HEK293T cells were incubated on ice with supernatant containing the serine variant of the rs7020782 SNP, the tyrosine variant of the rs7020782 SNP, or supernatant from HEK293T cells transfected with empty vector (MOCK) for 1 hour. A total of 800,000 cells/well were used. After incubation the cells were washed four times with 1% BSA in PBS and subsequently incubated with primary antibody against PAPP-A (two different antibodies were used: mAb 1/41^44^ and PA6^20^, both at a concentration of 10 µg/ml) for 30 min on ice. The cells were washed three times with 1% BSA in PBS and incubated with Alexa Fluor TM 488 goat anti mouse (Invitrogen A11029, 1:300) for 43 min on ice. After three washes, the cells were suspended and fixated in PBS with 2% paraformaldehyde and analysed on a Gallios Flow Cytometer. The 488 nm laser for excitation and FL1 (525/50 bandpass filter) detector for emission were used on the flow cytometer. The time-of-flight versus peak height analysis of forward scatter (FS) was used to exclude cell doublets, and a total of approximately 10,000–14,000 cells were gated for analysis in the software program FlowJo (version 10.5.2).

### Western blotting

In order to investigate the ability of the two rs7020782 PAPP-A variants to form complexes with STC2 and proMBP Western blotting was performed. Supernatants harvested from the HEK293T cells transfected with either of the two rs7020782 PAPP-A constructs, STC2, proMBP, or with empty vector (MOCK) as a negative control was used for Western blotting as previously described^20^. To remove possible traces of endogenous STC2 present in the media containing recombinant PAPP-A, immunoprecipitation of the culture media was carried out by incubating (16 h at 4 °C) 1 ml culture media with 30 ul protein G-Sepharose 4 Fast Flow beads (GE Healthcare, Denmark) coupled to 2 mg/ml of murine monoclonal antibodies against STC2 (STC221^20^,) as described previously^20^. Supernatants with either of the PAPP-A variants were incubated with STC2 or proMBP for 0, 1, 2, 4, 8, 16, and 24 hours (h) or with empty vector (MOCK) for 0, 8, and 24 h at 37 °C. STC2, proMBP and PAPP-A were blotted onto PVDF membranes (Millipore) following separation by 3–8% SDS-PAGE. For PAPP-A/STC2 and PAPP-A/proMBP complex detection, the membranes were incubated with rabbit polyclonal anti-PAPP-A at 0.63 μg/ml in TST supplemented with 2% skim milk for 16 h at 20 °C^45^. Membranes were washed with TST and subsequently incubated with polyclonal swine anti-rabbit IgG-HRP (DAKO, P0217) in TST supplemented with 2% skim milk for 1 h at 20 °C. Blots were developed using enhanced chemiluminescence (ECL Prime, GE Healthcare). Images were captured using an ImageQuant LAS 4000 instrument (GE Healthcare) (ECL; Amersham Pharmacia Biotech).

The PageRuler™ Plus Prestained Protein Ladder, 10 to 250 kDa was used as marker (Catalog number: 26619, Thermo Scientific™).

### Proteinase assay

To measure the proteolytic activity of PAPP-A in the supernatants harvested from the HEK293T cells transfected with either of the two rs7020782 variants, a proteinase assay based on autoradiography was performed as previously described in detail^16^. Proteinase assays were performed by measuring the proteolytic cleavage of radiolabelled IGFBP-2, IGFBP-4, and IGFBP-5. Different PAPP-A concentrations were used in the three assays, which were optimized for the variants to achieve cleavage percentages below 30%, where a linear relationship between time and cleavage can be assumed, and the cleavage rates are hereby determined by the slopes^16^. For the IGFBP-2 cleavage assay the PAPP-A concentration was adjusted to 10 nM, for the IGFBP-4 assay PAPP-A concentration was adjusted to 150 pM and for the IGFBP-5 assay the PAPP-A concentration was adjusted 80 pM. Intact and cleaved IGFBPs were quantified with the ImageQuant TL 8.1 software (GE Healthcare).

Addition of increasing concentrations of STC1 was performed in a separate IGFBP-4 cleavage assay to examine if the inhibition of PAPP-A was different between the two rs7020782 PAPP-A variants. The STC1 concentrations used in the assay were: 0.001 nM, 0.0312 nM, 0.0625 nM, 0.125 nM, 0.25 nM, 0.5 nM, and 1.0 nM^19^.

### Statistical analysis

An unpaired t-test was used to test significant difference in cleavage rate of IGFBP-2, IGFBP-4, and IGFBP-5 between the two rs7020782 PAPP-A variants. The same model was used to test significant differences in STC1 inhibition of PAPP-A activity and the cell surface binding ability. The p-value was set to 0.05 to assess statistical significance.

## Supplementary information

Supporting materials

## Data Availability

Data generated during this study are made available in the supplementary files. Additional data generated and analysed during the current study are available from the corresponding author on reasonable request.

## References

[1] Spicer LJ (2004). Proteolytic degradation of insulin-like growth factor binding proteins by ovarian follicles: a control mechanism for selection of dominant follicles. Biol Reprod..

[2] Conover CA, Oxvig C, Overgaard M, Christiansen M, Giudice L (1999). Evidence that the insulin-like growth factor binding protein-4 protease in human ovarian follicular fluid is pregnancy associated plasma protein-a. J. Clin Endocrinol Metab..

[3] Bøtkjær JA (2015). Pregnancy-associated plasma protein a in human ovarian follicles and its association with intrafollicular hormone levels. Fertil Steril..

[4] DeChiara TM, Efstratiadis A, Robertson EJ (1990). A growth-deficiency phenotype in heterozygous mice carrying an insulin-like growth factor II gene disrupted by targeting. Nature..

[5] Dauber A (2016). Mutations in pregnancy-associated plasma protein a 2 cause short stature due to low igf-i availability. EMBO Mol Med..

[6] Lee WS, Kim J (2017). Insulin-like growth factor-1 signaling in cardiac aging. Biochim Biophys Acta. Mol Basis Dis..

[7] Le Roith D (2003). The insulin-like growth factor system. Exp Diabesity Res..

[8] Frasca F (1999). Insulin receptor isoform a, a newly recognized, high-affinity insulin-like growth factor ii receptor in fetal and cancer cells. Mol Cell Biol..

[9] Mohan S, Baylink DJ, Pettis JL (1996). Insulin-like growth factor (igf)-binding proteins in serum–do they have additional roles besides modulating the endocrine igf actions?. J. Clin Endocrinol Metab..

[10] Firth SM, Baxter RC (2002). Cellular actions of the insulin-like growth factor binding proteins. Endocr Rev..

[11] Allard JB, Duan C (2018). Igf-binding proteins: Why do they exist and why are there so many?. Front Endocrinol..

[12] Laursen LS, Sorensen KK, Andersen MH, Oxvig C (2007). Regulation of insulin-like growth factor bioactivity by sequential proteolytic cleavage of igf binding protein-4 and -5. Mol Endocrinol..

[13] Overgaard MT (2001). Pregnancy-associated plasma protein-a2 (papp-a2), a novel insulin-like growth factor-binding protein-5 proteinase. J. Biol Chem..

[14] Mohan S (2002). Adam-9 is an insulin-like growth factor binding protein-5 protease produced and secreted by human osteoblasts. Biochemistry..

[15] Oxvig C (2015). The role of papp-a in the igf system: location, location, location. J. Cell Commun Signal..

[16] Gyrup C, Oxvig C (2007). Quantitative analysis of insulin-like growth factor-modulated proteolysis of insulin-like growth factor binding protein-4 and -5 by pregnancy-associated plasma protein-a. Biochemistry..

[17] Wald N (1992). First trimester concentrations of pregnancy associated plasma protein a and placental protein 14 in down’s syndrome. BMJ..

[18] Overgaard MT (2000). Expression of recombinant human pregnancy-associated plasma protein-a and identification of the proform of eosinophil major basic protein as its physiological inhibitor. J. Biol Chem..

[19] Kløverpris S (2015). Stanniocalcin-1 potently inhibits the proteolytic activity of the metalloproteinase pregnancy-associated plasma protein-A. J. Biol Chem..

[20] Jepsen MR (2016). The proteolytic activity of pregnancy-associated plasma protein-a is potentially regulated by stanniocalcin-1 and -2 during human ovarian follicle development. Hum Reprod..

[21] Jepsen MR (2015). Stanniocalcin-2 inhibits mammalian growth by proteolytic inhibition of the Insulin-like growth factor axis. J. Biol Chem..

[22] Kristensen T, Oxvig C, Sand O, Møller NP, Sottrup-Jensen L (1994). Amino acid sequence of human pregnancy-associated plasma protein-a derived from cloned cDNA. Biochemistry.

[23] Overgaard MT (2003). Complex of pregnancy-associated plasma protein-A and the proform of eosinophil major basic protein. Disulfide structure and carbohydrate attachment. J. Biol Chem..

[24] Boldt HB (2004). The lin12-notch repeats of pregnancy-associated plasma protein-a bind calcium and determine its proteolytic specificity. J. Biol Chem..

[25] Norman DG (1991). Three-dimensional structure of a complement control protein module in solution. J. Mol Biol..

[26] Weyer K (2004). Cell surface adhesion of pregnancy-associated plasma protein-a is mediated by four clusters of basic residues located in its third and fourth ccp module. Eur J. Biochem..

[27] Laursen LS (2002). Cell surface targeting of pregnancy-associated plasma protein a proteolytic activity. Reversible adhesion is mediated by two neighboring short consensus repeats. J. Biol Chem..

[28] Marouli E (2017). Rare and low-frequency coding variants alter human adult height. Nature..

[29] Suzuki K (2006). Pregnancy-associated plasma protein-a polymorphism and the risk of recurrent pregnancy loss. J. Reprod Immunol..

[30] Martinetti M (2017). The immunosignature of mother/fetus couples in gestational diabetes mellitus: Role of hla-g 14 bp ins/del and papp-a a/c polymorphisms in the uterine inflammatory milieu. Dis Markers..

[31] Zhou S (2015). Correlation of single nucleotide polymorphisms in the pregnancy-associated plasma protein-a gene with carotid plaques. BMC Cardiovasc Disord..

[32] Wang H (2012). Genetic relationship between serum pregnancy-associated plasma protein-a gene polymorphism and ischemic cerebrovascular disease in a northern han chinese population. Neural Regen Res..

[33] Bøtkjær JA (2016). Effect of pregnancy-associated plasma protein-a (papp-a) single-nucleotide polymorphisms on the level and activity of papp-a and the hormone profile in fluid from normal human small antral follicles. Fertil Steril..

[34] Database of Single Nucleotide Polymorphisms (dbSNP). Bethesda (MD): National Center for Biotechnology Information, National Library of Medicine. dbSNP accession: {NP_002572.2}, (dbSNP Build ID: {rs7020782}). Available from: http://www.ncbi.nlm.nih.gov/SNP/.

[35] Weyer K (2007). A substrate specificity-determining unit of three lin12-notch repeat modules is formed in trans within the pappalysin-1 dimer and requires a sequence stretch c-terminal to the third module. J. Biol Chem..

[36] Smith GC (2002). Early pregnancy levels of pregnancy-associated plasma protein a and the risk of intrauterine growth restriction, premature birth, preeclampsia, and stillbirth. J. Clin Endocrinol Metab..

[37] Baumgarten SC (2014). IGF1R signaling is necessary for fsh-induced activation of akt and differentiation of human cumulus granulosa cells. J. Clin Endocrinol Metab..

[38] Dugoff L (2004). First-trimester maternal serum papp-a and free-beta subunit human chorionic gonadotropin concentrations and nuchal translucency are associated with obstetric complications: a population-based screening study (the faster trial). Am J. Obstet Gynecol..

[39] Giorgetti C (2013). Multivariate analysis identifies the estradiol level at ovulation triggering as an independent predictor of the first trimester pregnancy-associated plasma protein-a level in ivf/icsi pregnancies. Hum Reprod..

[40] Kirkegaard I, Henriksen TB, Uldbjerg N (2011). Early fetal growth, papp-a and free β-hcg in relation to risk of delivering a small-for-gestational age infant. Ultrasound Obstet Gynecol..

[41] Bahceci M, Ulug U, Turan E, Akman MA (2007). Comparisons of follicular levels of sex steroids, gonadotropins and insulin like growth factor-1 (IGF-1) and epidermal growth factor (EGF) in poor responder and normoresponder patients undergoing ovarian stimulation with gnrh antagonist. Eur J. Obstet Gynecol Reprod Biol..

[42] Conover CA, Oxvig C (2018). PAPP-A and cancer. J Mol Endocrinol..

[43] Overgaard MT (2004). Inhibition of proteolysis by the proform of eosinophil major basic protein (prombp) requires covalent binding to its target proteinase. FEBS Lett..

[44] Mikkelsen JH, Steffensen LB, Oxvig C (2014). Development of a recombinant antibody towards papp-a for immunohistochemical use in multiple animal species. J. Immunol Methods..

[45] Oxvig C, Sand O, Kristensen T, Kristensen L, Sottrup-Jensen L (1994). Isolation and characterization of circulating complex between human pregnancy-associated plasma protein-a and proform of eosinophil major basic protein. Biochim Biophys Acta..

