# Author Correction: A common variant of the pregnancy-associated plasma protein-A (PAPPA) gene encodes a protein with reduced proteolytic activity towards IGF-binding proteins

**DOI:** 10.1038/s41598-019-53957-x

**Published:** 2019-11-21

**Authors:** J. A. Bøtkjær, P. R. Noer, Claus Oxvig, Claus Yding Andersen

**Affiliations:** 1 Laboratory of Reproductive Biology, The Juliane Marie Centre for Women, Children and Reproduction, Rigshospitalet, Copenhagen University Hospital, Copenhagen University, Copenhagen, DK-2100 Denmark; 2 Department of Molecular Biology and Genetics, University of Aarhus, Aarhus, DK-8000 Denmark

Correction to: *Scientific Reports* 10.1038/s41598-019-49626-8, published online 13 September 2019

In the original version of this Article, the authors Jane Alrø Bøtkjær and Pernille Rimmer Noer were incorrectly indexed. This error has now been corrected.

