# Supplementary material for: A common variant of the pregnancy-associated plasma protein-A (PAPPA) gene encodes a protein with reduced proteolytic activity towards IGF-binding proteins
Source: Sci Rep. 2019 Sep 13;9:13231. doi: 10.1038/s41598-019-49626-8 (PMC6744435; doi:10.1038/s41598-019-49626-8)
Supplement: Supplementary file 1 — Supporting materials [file 41598_2019_49626_MOESM1_ESM.pdf]

Supporting materials regarding the manuscript entitled: *"A common variant of the pregnancy-associated plasma protein-A (PAPPA) gene encodes a protein with reduced proteolytic activity towards IGF-binding proteins"*

Authors: Jane Alrø Bøtkjær, Pernille Rimmer Noer, Claus Oxvig, Claus Yding Andersen

Supporting materials 1:

Supplementary figure 1.1 Histograms obtained from flow cytometry. The fluorescence intensity on a log scale measured on a flow cytometer are displayed. Geometric means of the fluorescence are found in the table below the histograms for each sample, where a total of approximately 10,000-14,000 cells were gated for analysis in the software program FlowJo (version 10.5.2).
Red histograms: fluorescence intensity measured from HEK293T cells incubated with supernatants containing the serine variant (rPA_1144(Ser)). Blue bars: fluorescence intensity measured from HEK293T cells incubated with supernatants containing the tyrosine variant (rPA_1144(Tyr)). Black bars: fluorescence intensity measured from HEK293T cells incubated with supernatants transfected with empty vector (MOCK). Different PAPP-A monoclonal antibodies were tested: a) PA6 and b) mAb 1/41 (described in the manuscript). Experiments were completed in triplicate.

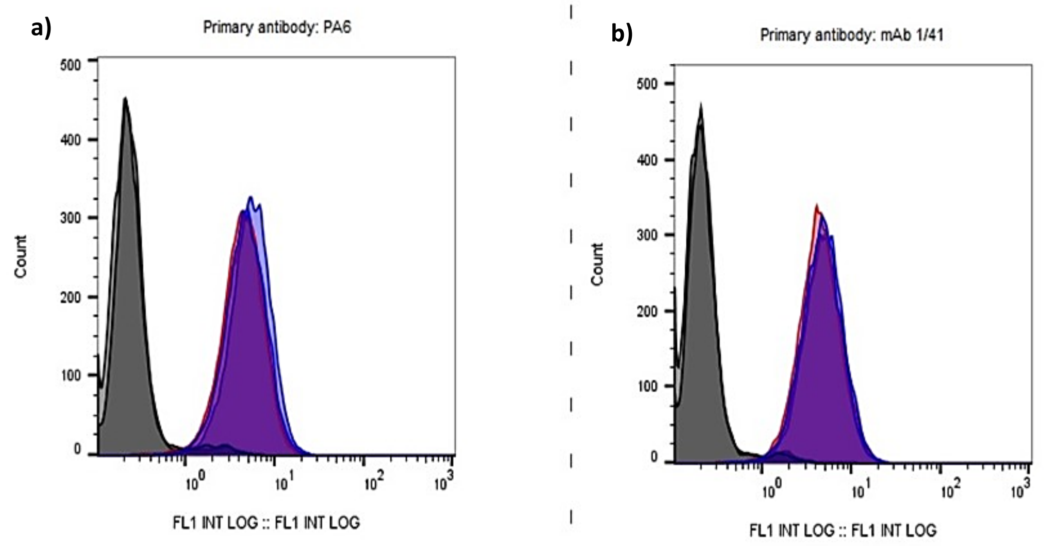

	Sample Name	Subset Name	Count	Geometric Mean : FL1 INT LOG
(blue)	171101_F3 00025763 248.LMD	Singlets	11294	4,50
(black)	171101_E3 00025762 247.LMD	Singlets	11759	0,24
(red)	171101_D3 00025761 246.LMD	Singlets	10877	4,21
(blue)	171101_C3 00025760 245.LMD	Singlets	11787	5,09
(black)	171101_B3 00025759 244.LMD	Singlets	10822	0,26
(red)	171101_A3 00025758 243.LMD	Singlets	11629	4,24

	Sample Name	Subset Name	Count	Geometric Mean : FL1 INT LOG
(blue)	171101_F4 00025770 255.LMD	Singlets	11350	4,37
(black)	171101_E4 00025769 254.LMD	Singlets	10797	0,23
(red)	171101_D4 00025768 253.LMD	Singlets	11667	4,15
(blue)	171101_C4 00025767 252.LMD	Singlets	11238	4,68
(black)	171101_B4 00025766 251.LMD	Singlets	11858	0,23
(red)	171101_A4 00025765 250.LMD	Singlets	10915	4,49

Supplementary figure 1.2 Flow cytometry experiments with various concentrations of PAPP-A.

We have assessed the cell adhesion assay with various and lower concentrations of PAPP-A to avoid saturation. These flow cytometry data do not show significant differences between the PAPP-A variants in their ability to adhere to the HEK293T cells.
Data are geometric mean of the fluorescence intensity measured on a flow cytometer relative to MOCK (±SD). The fluorescence intensity measured from HEK293T cells incubated with supernatants containing either the serine variant (PA(Ser): red bars) or the tyrosine variant (PA(Tyr): blue bars) of the rs7020782 PAPP-A SNP relative to MOCK (no PAPP-A) are shown. Experiments were tested with two different primary antibodies against PAPP-A (PA6 and mAb 1/41 antibody).

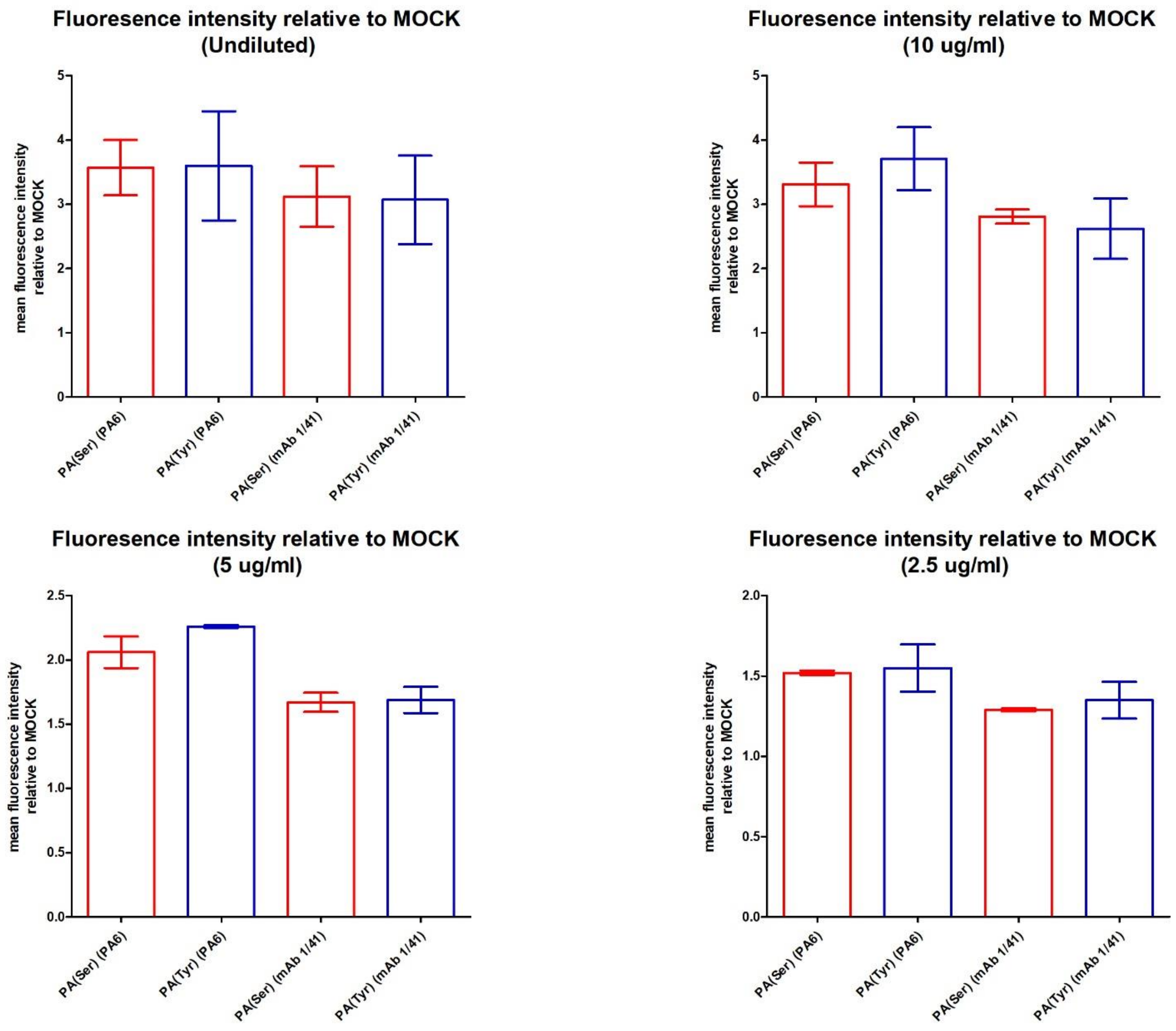

Supporting materials 2:

Supplementary figure 2.1 Full-length Western blots. Supernatants containing PAPP-A with either the serine (PA1144**S**) or the tyrosine (PA1144**Y**) variant of the rs7020782 SNP were incubated with STC2 (a and b) or proMBP (c and d) and formed covalent complexes over time (PAPP-A was incubated with STC2 or proMBP for 0, 1, 2, 4, 8, 16, and 24 hours (h) at 37°C). These protein complexes were separated by non-reducing SDS-page and visualized with Western blotting using a primary antibody against PAPP-A. A high molecular-weight band appears when complexes are formed (> 400kDa PAPP-A dimer). Supernatants from HEK293T cells co-transfected with both PAPP-A and STC2 or PAPP-A and proMBP were included in the Western blot as positive controls (co-trans) showing complexes exclusively.
When incubating the two rs7020782 PAPP-A variants with supernatant from transfected cells with empty vector (MOCK) over time no complex formation was observed (e).
PA(Ser): supernatant from cells transfected with the serine variant (PA1144**S**) and PA(Tyr): supernatant from cells transfected with the tyrosine variant (PA1144**Y**). PA(Ser) and PA(Tyr) show PAPP-A dimers solely.
The PageRuler™ Plus Prestained Protein Ladder, 10 to 250 kDa was used as marker (M) (Catalog number: 26619, Thermo Scientific™).

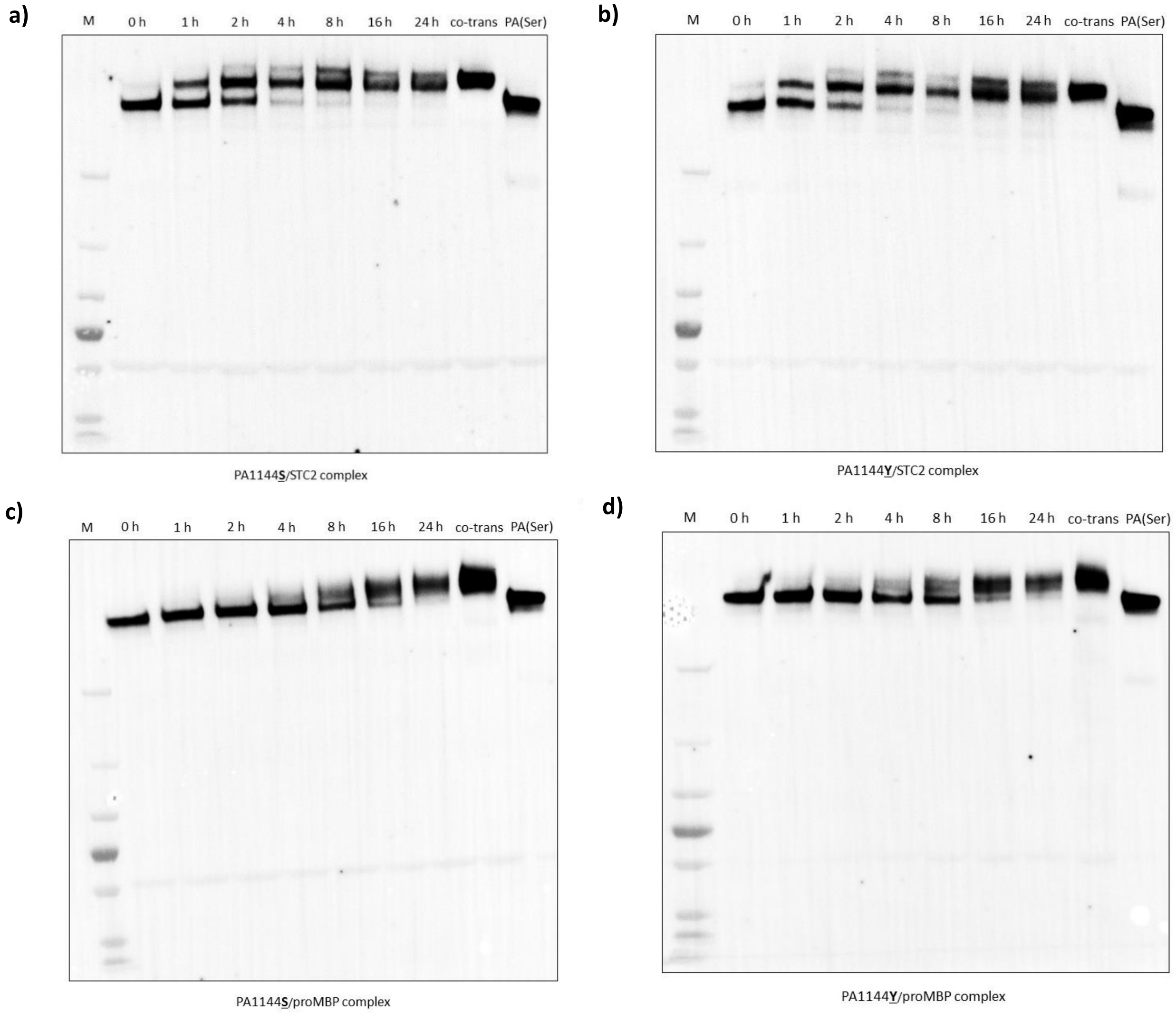

e)

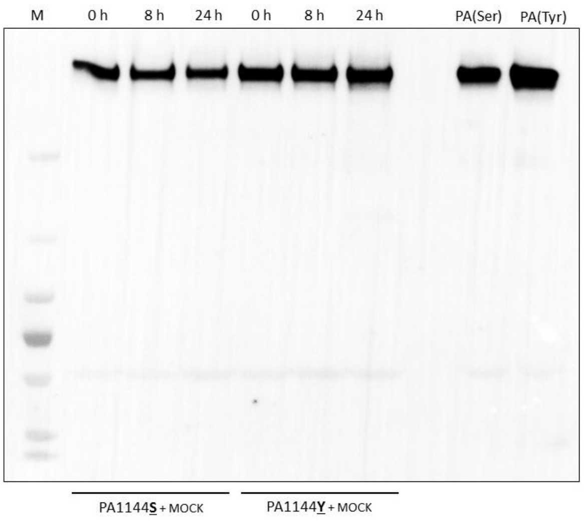

Supplementary figure 2.2 Replication of Western Blots. The following full-length Western Blots show a replication of the previous Western Blots shown in supplementary figure 2.1 where supernatants containing PAPP-A with either the serine (PA1144S) or the tyrosine (PA1144Y) variant of the rs7020782 SNP were incubated with STC2 (a and b) or proMBP (c and d) and formed covalent complexes over time. The PageRuler™ Plus Prestained Protein Ladder, 10 to 250 kDa was used as marker (M) (Catalog number: 26619, Thermo Scientific™). For further details see supplementary figure 2.1.

a)

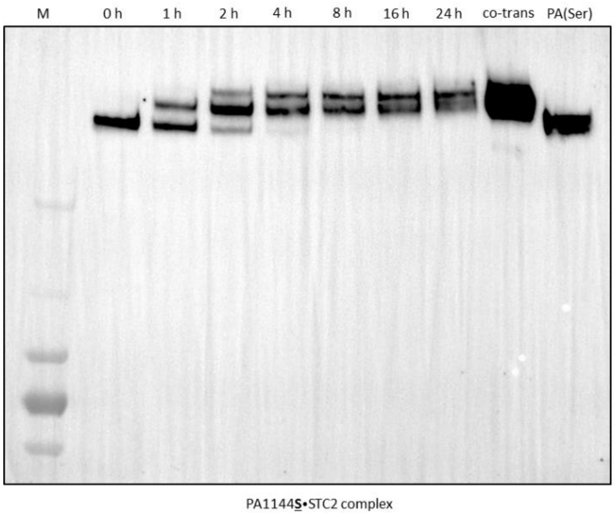

c)

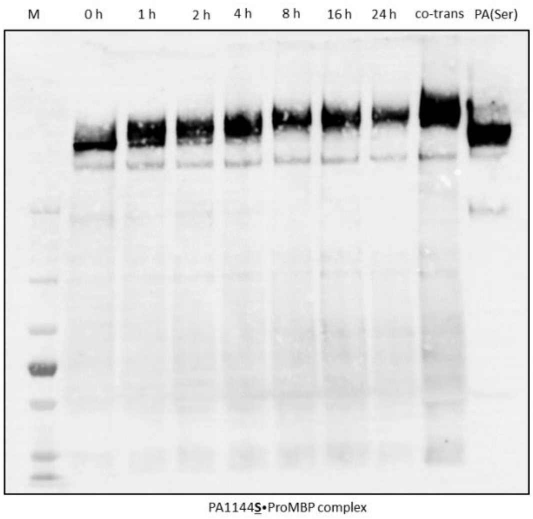

b)

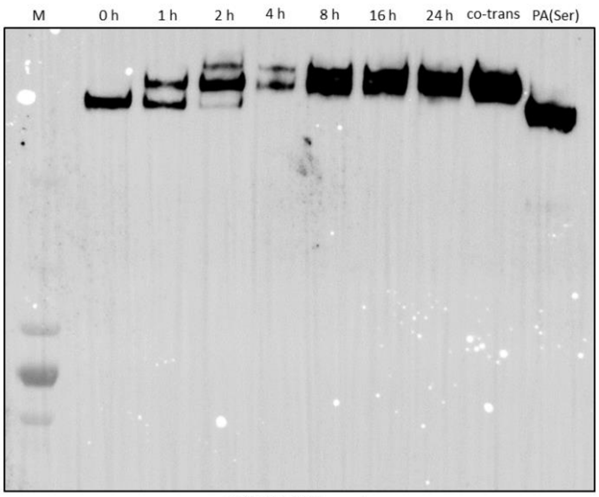

PA1144Y•STC2 complex

d)

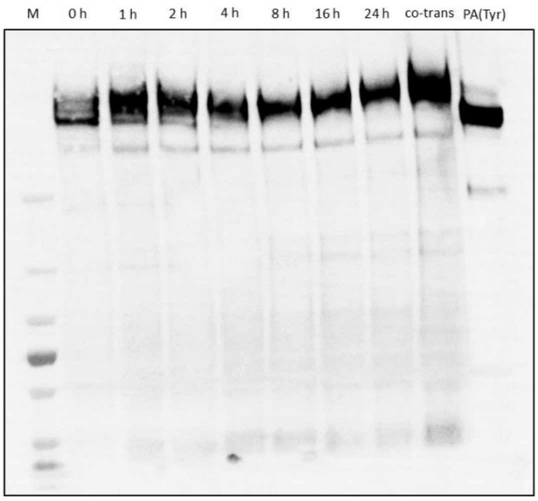

PA1144Y•ProMBP complex

Supporting materials 3.

Supplementary figure 3. Gel pictures from proteinase assays. To measure the proteolytic activity of PAPP-A in the supernatants harvested from the HEK293T cells transfected with either of the two rs7020782 variants, a proteinase assay based on autoradiography was performed. **3A)** Supernatants from HEK293T cells transfected with the two PAPP-A rs7020782 variants (serine: PA1144**S** and tyrosine: PA1144**Y**) were incubated with IGFBP-2 (upper panel), IGFBP-4 (mid panel), or IGFBP-5 (lower panel) at 37°C. Cleavage reactions were stopped at different time-points and substrate and cleavage products were separated by 12% SDS PAGE and subsequently visualized by autoradiography using a phosphor screen (Molecular Dynamics) and a Typhoon imaging system (GE Healthcare). Examples of these are shown in the following pictures (**3A**). Intact and cleaved IGFBPs were quantified with the ImageQuant TL 8.1 software (GE Healthcare).
Addition of increasing concentrations of STC1 was performed in a separate IGFBP-4 cleavage assay to examine if the inhibition of PAPP-A was different between the two rs7020782 PAPP-A variants (**3B**). The STC1 concentrations used in the assay were: S1: 1.0 nM, S2: 0.5 nM, S3: 0.25 nM, S4: 0.125 nM, S5: 0.0625 nM, and S6: 0.0312 nM. S0: no STC1.
Cleavage reactions were stopped at 3 time-points (15 min, 30 min, and 45 min) for each rs7020782 variant and an example is shown in **3B)** with a gel picture of cleavage reactions after 45 min for the tyrosine variant: PA1144**Y**.

3A)

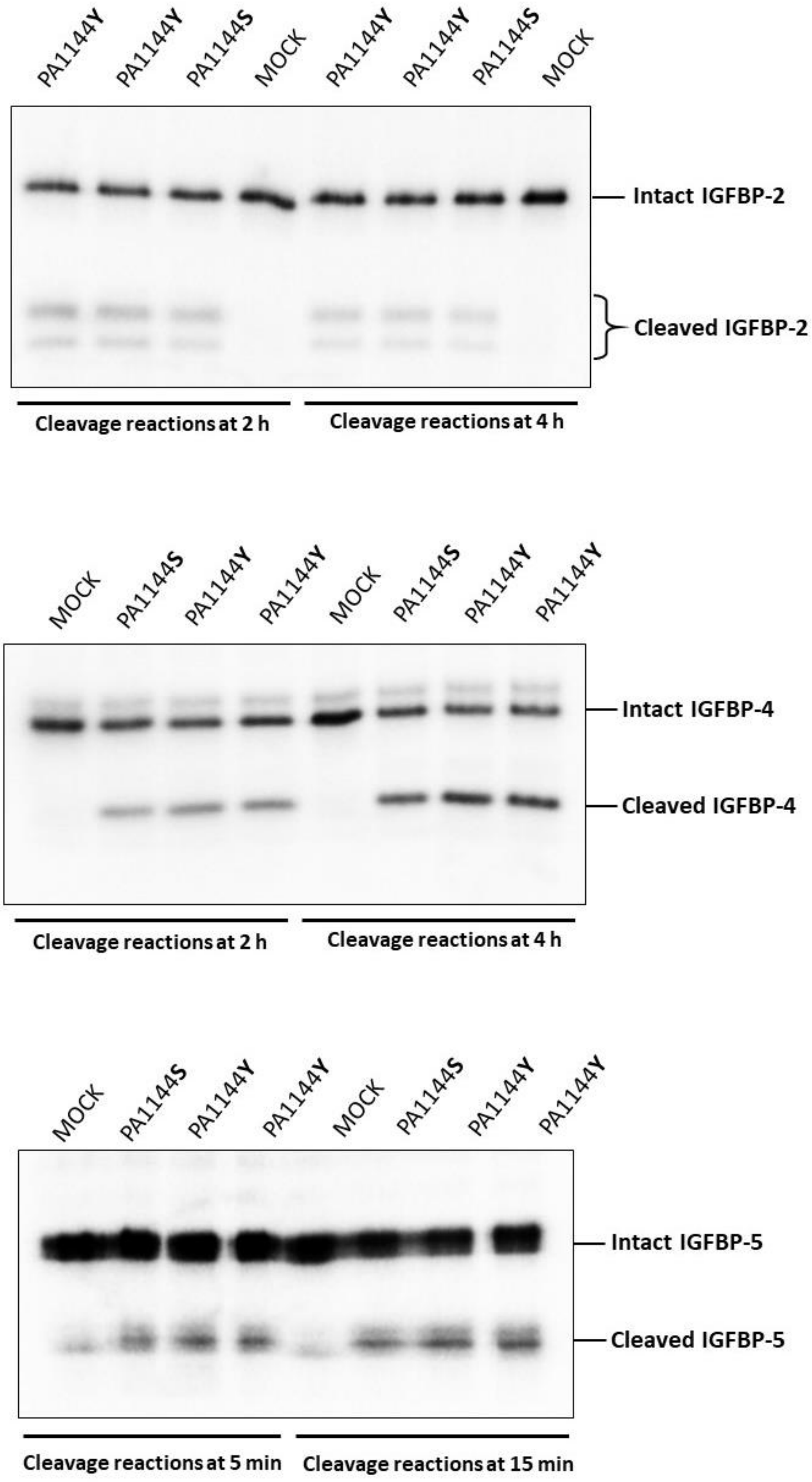

PA1144Y
PA1144Y
PA1144S
MOCK
PA1144Y
PA1144Y
PA1144S
MOCK
Intact IGFBP-2
Cleaved IGFBP-2
Cleavage reactions at 2 h
Cleavage reactions at 4 h
MOCK
PA1144S
PA1144Y
PA1144Y
MOCK
PA1144S
PA1144Y
PA1144Y
Intact IGFBP-4
Cleaved IGFBP-4
Cleavage reactions at 2 h
Cleavage reactions at 4 h
MOCK
PA1144S
PA1144Y
PA1144Y
MOCK
PA1144S
PA1144Y
PA1144Y
Intact IGFBP-5
Cleaved IGFBP-5
Cleavage reactions at 5 min
Cleavage reactions at 15 min

3B)

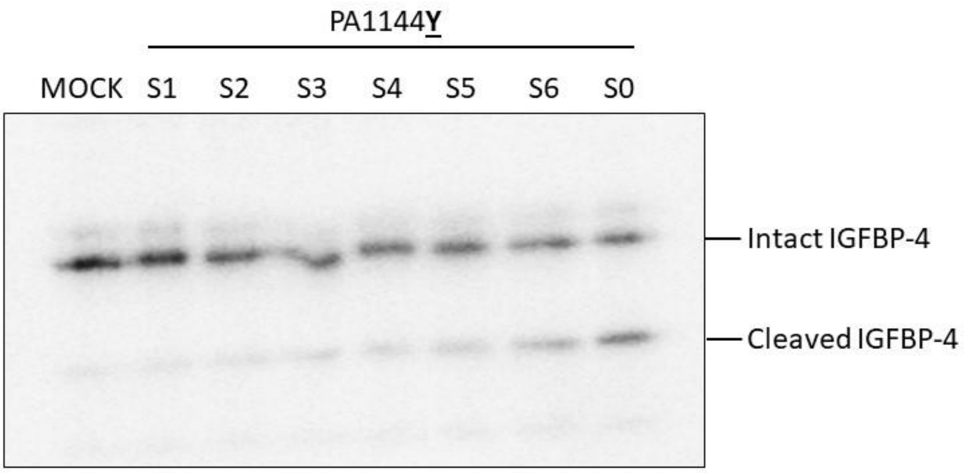

Cleavage reactions at 45 min
